# Neonatal sepsis due to glycopeptide resistant *Enterococcus faecium* from colonized maternal gut- rare case evidence

**DOI:** 10.1186/s13756-019-0490-x

**Published:** 2019-02-08

**Authors:** Supram Hosuru Subramanya, Rajesh Amberpet, Dinesh Chaudhary, Niranjan Nayak, Shashiraja Padukone, Indira Bairy, Shishir Gokhale

**Affiliations:** 1 Department of Medical Microbiology, Manipal College of Medical Sciences, Pokhara, Nepal; 2 Melaka Manipal Medical College, Manipal, India; 3 Jawaharlal Institute of Postgraduate Medical Education & Research, Puducherry, India

**Keywords:** VRE, Neonatal Sepsis, Gut colonization

## Abstract

**Background:**

Vancomycin-resistant enterococcal infections in the neonatal ICU are growing global problems. We report a case of neonatal septicemia by multidrug-resistant vancomycin-resistant *Enterococcus faecium* (VRE), the source of infection being the mother’s gut.

**Case presentation:**

A newborn male child admitted to the neonatal intensive care unit (NICU) was diagnosed to have mild meconium aspiration syndrome, early onset neonatal septicemia, and bacteremia by multidrug and vancomycin-resistant *Enterococcus faecium*. Screening of gut flora of the baby and the mother were carried out to trace the source of infection. Stool cultures of the mother and the baby yielded Vancomycin-Resistant *Enterococcus faecium.* All three isolates of *Enterococcus faecium* had similar antibiogram, harbored the *van*A gene and similar pulsed-field gel electrophoresis pattern. Baby responded to the 1 week therapy with oral linezolid suspension 20 mg/kg/day, 1 ml/t.d.s. No VRE was isolated from baby on a repeat stool culture 1 week after the linezolid therapy. He was discharged with the advice for the continuance of linezolid for seven more days.

**Conclusion:**

Isolation of MDR-VRE from the blood culture of the baby and stool specimens of the mother and the baby with the same antibiogram profile and clonal similarities reveals that maternal gut colonization was responsible for neonatal sepsis. Optimal infection control measures and the development of guidelines for monitoring VRE colonization in pregnant women might be useful in reducing the occurrence of neonatal sepsis.

**Electronic supplementary material:**

The online version of this article (10.1186/s13756-019-0490-x) contains supplementary material, which is available to authorized users.

## Background

*Enterococci,* regarded as harmless intestinal commensals of little clinical significance, over the past few decades have evolved as an important nosocomial pathogen [1]. This was mainly attributed to its acquisition of resistance to multiple antibiotics [2]. Vancomycin-resistant enterococcal infections in the neonatal ICUs are growing global problems. Neonatal infections due to Vancomycin-Resistant *Enterococcus* (VRE) could be by way of vertical transmission from the mother’s gastrointestinal tract or skin or vaginal canal or even could be acquired horizontally as cross-infection from the hospital environment [3, 4]. Whereas, early onset neonatal sepsis happens invariably due to pathogens acquired from the amnion or birth canal [4]; the origin of late-onset cases that occur beyond 7 days of age is less well established. Not much is known about the source for the transmission of this organism in causing infection in the neonates. Thus, we report a case of early onset neonatal septicemia by VRE, probably acquired from the mother.

## Case presentation

A male newborn child was admitted to the neonatal intensive care unit (NICU) because of vomiting and nasal flaring, immediately after birth. The baby weighed 3.1 kg and was born by normal vaginal delivery to a 27-year primigravida at 42 weeks of gestation. The Apgar scores were 7/10 at 1 min and 8/10 at 5 min. The baby was meconium stained, with a heart rate of 172/min, respiratory rate 74/min, temperature 98.2 °F, and SpO_2_ 88%. He was diagnosed to have early-onset neonatal sepsis. There was no history of antibiotic treatment, vaginal discharge or premature rupture of membrane or any maternal febrile episode during the antenatal period. The baby was shifted to NICU, and was empirically managed with IV (intravenous) ampicillin (100 mg/kg/dose, IV t.d.s), gentamicin (5 mg/kg/per, IV o.d), oxygen inhalation and intravenous infusion of dextrose saline.

The baby’s white blood cells count was 14,500/mm^3^ with 83% neutrophils, 17% lymphocytes. His hemoglobin was 15.2 g/dl; platelet count was 250,000/mm^3^. Blood biochemistry revealed Na^+^ 143 meq/L, K^+^ 4.6 meq/L, urea 18 mg/dl, creatinine 0.6 mg/dl and Ca^++^ 9.6 g/dl. CRP was 48 mg/L. Neonate’s blood sent for culture on day 1 of admission, grew *Enterococcus faecium* identified by conventional techniques [5]. Antibiotic susceptibility as determined by the Kirby-Bauer disc diffusion method [6] revealed the isolate to be resistant to penicillin (10 units), erythromycin (15 μg), gentamicin (10 μg), ciprofloxacin (5 μg), vancomycin (30 μg) and teicoplanin (30 μg), but susceptible to linezolid (30 μg) and chloramphenicol (30 μg). The isolate possessed high-level resistance to vancomycin, (MIC = 128 μg/ml) and teicoplanin (≥256 μg/ml) as tested by E test. On day 3, he had an episode of seizure which was managed with intravenous phenytoin 20 mg/kg stat, followed by a maintenance dose of 5 mg/kg/day b.d until convulsions were controlled. Thereafter, he was stabilized on phenobarbitone (5 mg/kg/day/o.d).

Based upon the above, the case was diagnosed as mild meconium aspiration syndrome with early-onset neonatal VRE septicemia. The antibiotic therapy was switched over to suspension linezolid 20 mg/kg/day, 1 ml/t.d.s (100 mg/5 ml) orally in view of the antibiotic susceptibility report.

VRE was not isolated from the environment of NICU during this period by microbiology surveillance. Surveillance was conducted by collecting samples from different sites of the NICU either in direct or indirect contact with the baby. These were feeding tubes, oxygen masks, linen and beddings, door handles, wash basins and, personal belongings such as the stethoscope and mobile phones of attending doctors and nurses, and hands of all health-care personnel.

For estimating gut colonization of both the baby and the mother and maternal vaginal colonization, we performed stool cultures (semi-quantitative) of both and vaginal swab culture of the mother. Stool specimens of the mother and the baby yielded heavy growth of Vancomycin-Resistant *Enterococcus faecium* with similar antibiogram profile. The antibiotic sensitivity patterns of the stool isolates were exactly similar to the isolate from the neonatal blood. No VRE was isolated from a maternal high vaginal swab. All the three isolates (both isolates from the stool and one from the neonatal blood) harbored *van*A gene as detected by polymerase chain reaction (PCR) assay [7] (Additional file 1: Figure S1). Typing of the isolates was done by pulsed-field gel electrophoresis (PFGE) as previously described [8] including one characterized clinical isolate and results were inspected visually. The PFGE pattern of all three isolates confirmed these to be clonally related (Additional file 1: Figure S1). The limitation of the PFGE analysis was that the results were not confirmed by digesting with a secondary enzyme. No VRE was isolated from baby on a repeat stool culture 1 week after the linezolid therapy. He was discharged with the advice for the continuance of linezolid for seven more days. At the time of discharge, all vital functions of the baby were within normal limits.

## Discussion

Of all the species of *Enterococci*, *E faecalis* and *E faecium* are the commonest human pathogens. Serious enterococcal infections are often difficult to treat because the organism exhibits intrinsic resistance to various antibiotics including penicillins, cephalosporins, and sometimes to aminoglycosides and clindamycin and other lincosamides [3]. The recent emergence of multidrug-resistant (MDR) *Enterococci,* especially VRE has provoked the occurrence of several nosocomial outbreaks worldwide [9].

*Enterococci* are among the first few bacteria that colonize the neonatal gastrointestinal tract from mother’s vaginal and gastrointestinal flora during delivery, or via ingestion of breast milk or through close association with the mother [10]. Although, we did not try to explore the exact route of transmission of the organism in the present case, the two possible routes of spread [11–13], in our view, may be (i) from maternal gut to maternal bloodstream, then transplacentally to the fetus, or (ii) maternal gut to neonatal oral cavity during parturition, spreading from there to neonatal blood. Since it is a case of early onset neonatal sepsis, the time frame suggests the first route be the most plausible one, although such pathways are not well documented among *Enterococcus*. Emphasizing further on this, isolating VRE from the neonate’s blood, mother’s and neonate’s gut, with similar antibiogram and similar PFGE pattern supports the proposition of the mother is the source of infection.

Employing PCR ribotyping, Kaushal S and colleagues [14], from India reported *van*A genotypes of *E faecalis* in the stool and blood of a neonate with septicemia and stated that gastrointestinal carriage could be the possible source of sepsis. Carl MA, et al. [15] suggested that vertically transmitted gram negative bacilli (GNB) and Group B *Streptococci* during vaginal delivery, colonize the neonatal gastrointestinal tract with the subsequent episode of late-onset septicemia. Das et al. [16], noticed that neonates with GNB in the gut had a consistently higher incidence of clinical sepsis than those without such colonization.

Our observation of similar PFGE patterns of the fecal and blood isolates substantiates strong association of maternal gut colonization with neonatal sepsis. The baby in the present case was diagnosed as mild meconium aspiration syndrome on clinical grounds. Bacterial diversity of meconium was assessed previously in preterm and term babies which had a profound effect on the health of the newborn, more so of the preterm babies [17]. We did not check meconium for sterility. Moles L, et al. [17] and Jiménez E et al. [18] proposed that fetal gut might not be sterile and that a part of the bacterial flora found in the meconium might not be of postnatal origin all the time. Besides, it was also postulated that many of the bacterial genera found in the meconium, including *Enterococcus* were common inhabitants of the maternal gut [19]. Transplacental migration of *E faecium* was demonstrated by Jiménez E et al. [18] in an experiment where the pregnant mice were fed with milk containing labeled *E faecium.* The meconium from the pups delivered by cesarean section yielded same labeled *E faecium*. The gut flora of the mother can spread to the neonate during the surgical procedure [20, 21], via trachea [22] and can lead to bacteremia.

Nevertheless, it is needless to emphasize that nosocomial sepsis following gut colonization in the neonates is of paramount clinical relevance, especially in developing countries, where inappropriate infection control measures prevail in most of the hospitals. The pathogen *Enterococcus* in the present case was resistant to multiple antibiotics including vancomycin and teicoplanin. In the face of increasing resistance rates and limited treatment options, prevention of multidrug-resistant *Enterococcus* infections is of utmost importance. Thus CDC recently recommended the use of all contact precautions like barrier nursing, hand washing etc. not only for carriers of MDR *Enterococcus* but for all MDR organisms in order to prevent the spread of these organisms in health care settings [23]. Some hospitals practiced routine screening and decolonization of MRSA carriers to prevent the subsequent spread of infections [24]. Contrast to this effective screening and decolonization of VRE carriers are not practiced in many hospitals as VRE is much less common invasive pathogens than MRSA and tends to impact a smaller subset of patients [25].

Notwithstanding the above, other researchers [3, 4, 14] too, reported high *van*A VRE carriage rate in the feces of patients admitted to ICUs. The predominance of *van*A genotype of *Enterococcus faecium* was previously documented in Asian countries such as Japan, Korea, China, and India [3, 9]. *Enterococcus* belonging to the *van*A type has become a cause of concern because of its high-level resistance to vancomycin, and high potential of transfer of this gene to other potent pathogens like *Staphylococcus aureus* [26].

## Conclusion

Isolation of MDR-VRE from stool specimens of both the mother and the baby with similar antibiogram and PFGE pattern reveals that maternal gut colonization might be responsible for neonatal sepsis. There is a need for implementation of strategies to prevent the dissemination of these pathogens from mother to neonate and among the newborn babies. Optimal infection control measures and the development of guidelines for the control of colonization by VRE in pregnant women might be useful in the reduction of neonatal sepsis.

## Additional file

Additional file 1: **Figure S1. (a)** Polymerase chain reaction amplification of *vanA* gene in *Enterococcus fecacieum* isolated from baby blood (lane D) and stool of the neonate (lane E) and mother (lane F). (Lane A: 100 bp ladder, B: positive control for vanA gene (732 bp), C: negative control). **(b)** Typing of the isolates: Strain relation according to PFGE (Lane 1: molecular marker) (PCR 20 bp Low Ladder, Sigma-Aldrich, USA). PFGE showed that both blood and stool isolates were pulso type A. (PNG 2020 kb)

## Abbreviations

CRP: C-reactive protein
IV: Intravenous
MDR: Multi Drug Resistant
NICU: Neonatal intensive care unit
PCR: Polymerase chain reaction
SPO2: Peripheral capillary oxygen saturation
t.d.s: Thrice a day
VRE: Vancomycin Resistant Enterococci
