# Supplementary figures and images for: Neonatal sepsis due to glycopeptide resistant Enterococcus faecium from colonized maternal gut- rare case evidence
Source: Antimicrob Resist Infect Control. 2019 Feb 8;8:29. doi: 10.1186/s13756-019-0490-x (PMC6368750; doi:10.1186/s13756-019-0490-x)

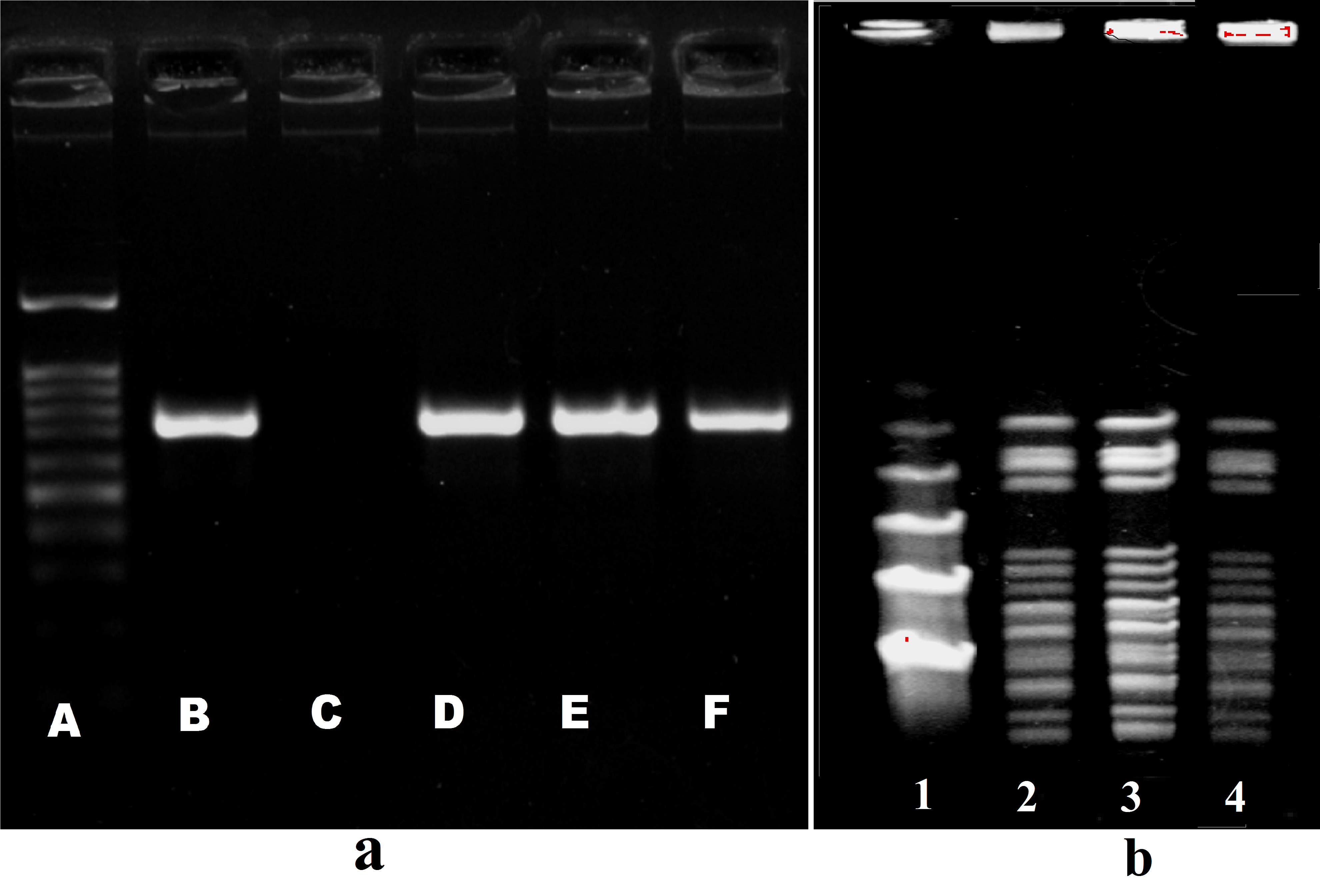

Supplement: Supplementary file 1 — Figure S1. (a) Polymerase chain reaction amplification of vanA gene in Enterococcus fecacieum isolated from baby blood (lane D) and stool of the neonate (lane E) and mother (lane F). (Lane A: 100 bp ladder, B: positive control for vanA gene (732 bp), C: negative control). (b) Typing of the isolates: Strain relation according to PFGE (Lane 1: molecular marker) (PCR 20 bp Low Ladder, Sigma-Aldrich, USA). PFGE showed that both blood and stool isolates were pulso type A. (PNG 2020 kb) [file 13756_2019_490_MOESM1_ESM.png]
